# Supplementary material for: Medication Logistics in Professional Homecare Organisations: An Assessment of the Practical Implementation of Regulations and Recommendations
Source: Nurs Rep. 2025 Sep 10;15(9):332. doi: 10.3390/nursrep15090332 (PMC12472274; doi:10.3390/nursrep15090332)
Supplement: Supplementary file 1 [file nursrep-15-00332-s001.zip › nursrep-3807640-Supplementary S1.pdf]

Supplementary Material S1

Inclusion and exclusion criteria for the title / abstract screening and the full-text screening of the scoping literature review

Inclusion criteria	Exclusion criteria
Professional home care services that support patients with their medication management or take over the management.	Outpatient services that do not go to the patient's home.<br>Non-professional home care services.
Mention of ordering, delivery or central storage of drugs	
All other types of publication	Conference abstracts, proceeding papers, protocols, reviews
Languages: German, English, French, Spanish, Italian	All other languages
