# Supplementary material for: Medication Logistics in Professional Homecare Organisations: An Assessment of the Practical Implementation of Regulations and Recommendations
Source: Nurs Rep. 2025 Sep 10;15(9):332. doi: 10.3390/nursrep15090332 (PMC12472274; doi:10.3390/nursrep15090332)
Supplement: Supplementary file 1 [file nursrep-15-00332-s001.zip › nursrep-3807640-Supplementary S2.pdf]

Supplementary Material S2

Scoping review – search string for the PubMed, Embase and CINAHL databases

Topic	MeSH-Terms	Database	Text words	Search string	Results
**Professional home care services**	Home Care Services<br>Home Care Agencies<br>Home Health Nursing	PubMed	Homecar*<br>Home car*<br>Home-based car*<br>Home support*<br>Domiciliary car*<br>Hospital at home<br>Community car*<br>Spitex<br>Home healthcare nursing<br>In-home healthcare services<br>In-home nursing services<br>Home-based nursing<br>At-home nursing<br>Home health care<br>Nursing care at home<br>Hospital at home	("Home Care Services"[Mesh] OR "Home Care Agencies"[Mesh] OR "Home Health Nursing"[Mesh] OR "Homecar*"[tiab] OR "Home car*"[tiab] OR "Home-based car*"[tiab] OR "Home support*"[tiab] OR "Domiciliary car*"[tiab] OR "Hospital at home"[tiab] OR "Community car*"[tiab] OR "Spitex"[tiab] OR "Home healthcare nursing"[tiab] OR "In-home healthcare services"[tiab] OR "In-home nursing services"[tiab] OR "Home-based nursing"[tiab] OR "At-home nursing"[tiab] OR "Home health care"[tiab] OR "Nursing care at home"[tiab] OR "Hospital at home"[tiab])	71'812

Topic	MeSH-Terms	Database	Text words	Search string	Results
**Medication management: medication logistics**	Medication Systems<br>Drug Storage<br>Task Performance and Analysis	PubMed	Drug storage<br>Medication management*<br>Medication administration*<br>Medicine management*"<br>Drug management*<br>Drug stock*<br>Drug order*<br>Medicine storag*<br>Medication storag*<br>Pharmaceutical storag*<br>Medication process<br>Medication use process<br>Stored medicines	("Medication Systems"[Mesh] OR "Drug Storage"[Mesh] OR "Task Performance and Analysis"[Mesh] OR "Drug Storage"[tiab] OR "Medication management*"[tiab] OR "Medication administration*"[tiab] OR "Medicine management*"[tiab] OR "Drug management*"[tiab] OR "Drug stock*"[tiab] OR "Drug order*"[tiab] OR "Medicine storag*"[tiab] OR "Medication storag*"[tiab] OR "Pharmaceutical storag*"[tiab] OR "Medication process"[tiab] OR "Medication use process"[tiab] OR "Stored medicines"[tiab])	61'622

Topic	Emtree-Terms	Database	Text words	Search string	Results
**Professional home care services**	Home care<br>Visiting nursing setting	Embase	Homecar*<br>Home car*<br>Home-based car*<br>Home support*<br>Domiciliary car*<br>Hospital at home<br>Community car*<br>Spitex<br>Home healthcare nursing<br>In-home healthcare services<br>In-home nursing services<br>Home-based nursing<br>At-home nursing<br>Home health care<br>Nursing care at home<br>Hospital at home	('Home care'/exp OR Visiting nursing setting/exp OR Homecar*:ti,ab OR 'Home car*':ti,ab OR 'Home-based car*':ti,ab OR 'Home support*':ti,ab OR 'Domiciliary car*':ti,ab OR 'Hospital at home':ti,ab OR 'Community car*':ti,ab OR Spitex:ti,ab OR 'Home healthcare nursing':ti,ab OR 'In-home healthcare services':ti,ab OR 'In-home nursing services':ti,ab OR 'Home-based nursing':ti,ab OR 'At-home nursing':ti,ab OR 'Home health care':ti,ab OR 'Nursing care at home':ti,ab OR 'Hospital at home':ti,ab)	45'565

Topic	Emtree-Terms	Database	Text words	Search string	Results
**Medication management: medication logistics**	Drug storage<br>Task performance	Embase	Drug storage<br>Medication management*<br>Medication administration*<br>Medicine management*"<br>Drug management*<br>Drug stock*<br>Drug order*<br>Medicine storag*<br>Medication storag*<br>Pharmaceutical storag*<br>Medication process<br>Medication use process<br>Stored medicines	('Drug storage'/exp OR 'Task performance'/exp OR 'Drug Storage':ti,ab OR 'Medication management*':ti,ab OR 'Medication administration*':ti,ab OR 'Medicine management*':ti,ab OR 'Drug management*':ti,ab OR 'Drug stock*':ti,ab OR 'Drug order*':ti,ab OR 'Medicine storag*':ti,ab OR 'Medication storag*':ti,ab OR 'Pharmaceutical storag*':ti,ab OR 'Medication process':ti,ab OR 'Medication use process':ti,ab OR 'Stored medicines':ti,ab)	190'072

Topic	Subject headings	Database	Text words	Search string	Results
**Professional home care services**	Home Health Care<br>Home Health Nurses	CINAHL	Homecar*<br>Home car*<br>Home-based car*<br>Home support*<br>Domiciliary car*<br>Hospital at home<br>Community car*<br>Spitex<br>Home healthcare nursing<br>In-home healthcare services<br>In-home nursing services<br>Home-based nursing<br>At-home nursing<br>Home health care<br>Nursing care at home<br>Hospital at home	((MH "Home Health Care+") OR (MH "Home Health Nurses+") OR (TI Homecar* OR AB Homecar*) OR (TI "Home car*" OR AB "Home car*") OR (TI "Home-based car*" OR AB "Home-based car*") OR (TI "Home support*" OR AB "Home support*") OR (TI "Domiciliary car*" OR AB "Domiciliary car*") OR (TI "Hospital at home" OR AB "Hospital at home") OR (TI "Community car*" OR AB "Community car*") OR (TI Spitex OR AB Spitex) OR (TI "Home healthcare nursing" OR AB "Home healthcare nursing") OR (TI "In-home healthcare services" OR AB "In-home healthcare services") OR (TI "In-home nursing services" OR AB "In-home nursing services") OR (TI "Home-based nursing" OR AB "Home-based nursing") OR (TI "At-home nursing" OR AB "At-home nursing") OR (TI "Home health care" OR AB "Home health care") OR (TI "Nursing care at home" OR AB "Nursing care at home") OR (TI "Hospital at home" OR AB "Hospital at home"))	64’830

Topic	Subject headings	Database	Text words	Search string	Results
**Medication management: medication logistics**	Medication Management<br>Drug Storage<br>Home Health Care Information Systems	CINAHL	Drug storage<br>Medication management*<br>Medication administration*<br>Medicine management*”<br>Drug management*<br>Drug stock*<br>Drug order*<br>Medicine storag*<br>Medication storag*<br>Pharmaceutical storag*<br>Medication process<br>Medication use process<br>Stored medicines	((MH "Medication Management+") OR (MH "Drug Storage+") OR (MH "Home Health Care Information Systems+") OR (TI "Drug Storage" OR AB "Drug Storage") OR (TI "Medication management*" OR AB "Medication management*") OR (TI "Medication administration*" OR AB "Medication administration*") OR (TI "Medicine management*" OR AB "Medicine management*") OR (TI "Drug management*" OR AB "Drug management*") OR (TI "Drug stock*" OR AB "Drug stock*") OR (TI "Drug order*" OR AB "Drug order*") OR (TI "Medicine storag*" OR AB "Medicine storag*") OR (TI "Medication storag*" OR AB "Medication storag*") OR (TI "Pharmaceutical storag*" OR AB "Pharmaceutical storag*") OR (TI "Medication process" OR AB "Medication process") OR (TI "Medication use process" OR AB "Medication use process") OR (TI "Stored medicines" OR AB "Stored medicines"))	10’505

Databases	Search-String: Professional home care services + Medication management: medication logistics	Results
**PubMed**	("Home Care Services"[Mesh] OR "Home Care Agencies"[Mesh] OR "Home Health Nursing"[Mesh] OR "Homecar*"[tiab] OR "Home car*"[tiab] OR “Home-based car*”[tiab] OR “Home support*”[tiab] OR "Domiciliary car*"[tiab] OR “Hospital at home”[tiab] OR “Community car*"[tiab] OR “Spitex"[tiab] OR “Home healthcare nursing”[tiab] OR “In-home healthcare services”[tiab] OR “In-home nursing services”[tiab] OR “Home-based nursing”[tiab] OR “At-home nursing”[tiab] OR “Home health care"[tiab] OR “Nursing care at home"[tiab] OR “Hospital at home"[tiab]) AND (“Medication Systems”[Mesh] OR “Drug Storage”[Mesh] OR “Task Performance and Analysis”[Mesh] OR "Drug Storage"[tiab] OR "Medication management*"[tiab] OR "Medication administration*"[tiab] OR "Medicine management*"[tiab] OR "Drug management*"[tiab] OR "Drug stock*"[tiab] OR "Drug order*"[tiab] OR "Medicine storag*"[tiab] OR “Medication storag*”[tiab] OR “Pharmaceutical storag*”[tiab] OR “Medication process"[tiab] OR “Medication use process"[tiab] OR “Stored medicines"[tiab])	637
**Embase**	('Home care’/exp OR Visiting nursing setting/exp OR Homecar*:ti,ab OR 'Home car*':ti,ab OR 'Home-based car*':ti,ab OR 'Home support*':ti,ab OR 'Domiciliary car*':ti,ab OR 'Hospital at home':ti,ab OR 'Community car*':ti,ab OR Spitex:ti,ab OR 'Home healthcare nursing':ti,ab OR 'In-home healthcare services':ti,ab OR 'In-home nursing services':ti,ab OR 'Home-based nursing':ti,ab OR 'At-home nursing':ti,ab OR 'Home health care':ti,ab OR 'Nursing care at home':ti,ab OR 'Hospital at home':ti,ab) AND ('Drug storage'/exp OR 'Task performance'/exp OR 'Drug Storage':ti,ab OR 'Medication management*':ti,ab OR 'Medication administration*':ti,ab OR 'Medicine management*':ti,ab OR 'Drug management*':ti,ab OR 'Drug stock*':ti,ab OR 'Drug order*':ti,ab OR 'Medicine storag*':ti,ab OR 'Medication storag*':ti,ab OR 'Pharmaceutical storag*':ti,ab OR 'Medication process':ti,ab OR 'Medication use process':ti,ab OR 'Stored medicines':ti,ab)	516

**CINAHL**	((MH "Home Health Care+") OR (MH "Home Health Nurses+") OR (TI Homecar* OR AB Homecar*) OR (TI "Home car*" OR AB "Home car*") OR (TI "Home-based car*" OR AB "Home-based car*") OR (TI "Home support*" OR AB "Home support*") OR (TI "Domiciliary car*" OR AB "Domiciliary car*") OR (TI "Hospital at home" OR AB "Hospital at home") OR (TI "Community car*" OR AB "Community car*") OR (TI Spitex OR AB Spitex) OR (TI "Home healthcare nursing" OR AB "Home healthcare nursing") OR (TI "In-home healthcare services" OR AB "In-home healthcare services") OR (TI "In-home nursing services" OR AB "In-home nursing services") OR (TI "Home-based nursing" OR AB "Home-based nursing") OR (TI "At-home nursing" OR AB "At-home nursing") OR (TI "Home health care" OR AB "Home health care") OR (TI "Nursing care at home" OR AB "Nursing care at home") OR (TI "Hospital at home" OR AB "Hospital at home")) AND ((MH "Medication Management+") OR (MH "Drug Storage+") OR (MH "Home Health Care Information Systems+") OR (TI "Drug Storage" OR AB "Drug Storage") OR (TI "Medication management*" OR AB "Medication management*") OR (TI "Medication administration*" OR AB "Medication administration*") OR (TI "Medicine management*" OR AB "Medicine management*") OR (TI "Drug management*" OR AB "Drug management*") OR (TI "Drug stock*" OR AB "Drug stock*") OR (TI "Drug order*" OR AB "Drug order*") OR (TI "Medicine storag*" OR AB "Medicine storag*") OR (TI "Medication storag*" OR AB "Medication storag*") OR (TI "Pharmaceutical storag*" OR AB "Pharmaceutical storag*") OR (TI "Medication process" OR AB "Medication process") OR (TI "Medication use process" OR AB "Medication use process") OR (TI "Stored medicines" OR AB "Stored medicines"))	472
**PubMed + Embase + CINAHL**	Total after deduplication:	1167
	After 1st screening (title / abstract):	45
	After 2nd Screening (full text):	10
