# Supplementary material for: Medication Logistics in Professional Homecare Organisations: An Assessment of the Practical Implementation of Regulations and Recommendations
Source: Nurs Rep. 2025 Sep 10;15(9):332. doi: 10.3390/nursrep15090332 (PMC12472274; doi:10.3390/nursrep15090332)
Supplement: Supplementary file 1 [file nursrep-15-00332-s001.zip › nursrep-3807640-Supplementary S3.pdf]

Supplementary Material S3

Inclusion criteria for the title / abstract screening and the full-text screening of the scoping literature review

Dear professional homecare organisation employees

Thank you very much for taking the time to complete the following survey on medication logistics in professional homecare organisations.

I am a pharmacy student at the University of Basel and I am researching medication logistics in professional homecare organisations for my five-month master's thesis, with a focus on the ordering and storage of medications.

This survey aims to find out how your professional homecare organisation handles medication logistics, whether your organisation has issued guidelines on this (e.g. standard operating procedures in the quality management system) and what obstacles you see in implementing them. The aim of the master's thesis is to conduct a status survey among professional homecare organisations in German-speaking Switzerland with regard to medication logistics.

You can complete the survey from Friday, 22 March 2024, to Wednesday, 10 April 2024. It will take a maximum of 30 minutes to answer the questions.

Your answers will be evaluated anonymously and it will not be possible to identify your professional homecare organisation. We may publish the results in anonymised form. By completing the questionnaire, you agree to this process.

If your professional homecare organisation has several locations and medication logistics are not handled in the same way at all locations, please forward the survey to the respective locations. Please do not complete the survey on behalf of and for several professional homecare organisations in your canton. Please answer all questions completely and in order, if possible. Do not jump from question to question, as the order of the questions changes depending on your answers. For the sake of simplicity, I will refrain from mentioning all genders below, but all genders are always meant.

I am available to answer any questions you may have.

Translated with DeepL.com (free version) (https://www.deepl.com/de/translator)
